# Estrogen-Related Factors in the Frontal Lobe of Alzheimer’s Disease Patients and Importance of Body Mass Index

**DOI:** 10.1038/s41598-017-00815-3

**Published:** 2017-04-07

**Authors:** Naoko Honma, Shigehira Saji, Tetuo Mikami, Noriko Yoshimura, Seijiro Mori, Yuko Saito, Shigeo Murayama, Nobuhiro Harada

**Affiliations:** 1 Department of Pathology, Toho University School of Medicine, Omori-Nishi 5-21-16, Ota-ku, Tokyo 143-8540 Japan; 2 Department of Medical Oncology, Fukushima Medical University, Hikariga-oka 1, Fukushima City, Fukushima 960-1295 Japan; 3 Department of Biochemistry, Fujita Health University School of Medicine, Dengakugakubo 1-98, Kutsukake-cho, Toyoake 470-1192 Japan; 4 Department of Internal Medicine, Tokyo Metropolitan Geriatric Hospital, Sakaecho 35-2, Itabashi-ku, Tokyo 173-0015 Japan; 5 Department of Pathology and Laboratory Medicine, National Center Hospital, National Center of Neurology and Psychiatry, Ogawa-Higashi 4-1-1, Kodaira, Tokyo 187-8551 Japan; 6 Department of Neuropathology, Tokyo Metropolitan Institute of Gerontology, Sakaecho 35-2, Itabashi-ku, Tokyo 173-0015 Japan

## Abstract

Estrogens play a physiologically important role in the brain, but controversies exist regarding the association between Alzheimer’s disease (AD) and estrogens. Estrogen-related factors were comprehensively examined in frontal lobe tissues from autopsied AD patients, and compared with controls. Concentrations of estrogens, expression of estrogen receptors (ERs), and estrogen-metabolizing enzymes (EMEs) which are important for determining the peripheral estrogen concentrations, were examined using liquid chromatography tandem mass spectrometry, immunohistochemistry, and quantitative real-time PCR, respectively. Body mass index (BMI), known to correlate with the serum estrogen concentrations, was also taken into consideration. There were no significant differences in estrogen concentrations or each EME level between the two groups in both the cortex and white matter, whereas glial nuclear ER-β expression was significantly lower in white matter from the AD group than the control group (Allred score, 3.2 ± 0.3 and 6.5 ± 0.3, respectively. *P* < 0.0001). Estrogen concentrations were found to closely correlate with BMI, particularly in controls. ER-β loss in the white matter from the AD group suggests the necessity of studying the effects of estrogens on glias as well as neurons in the etiology of AD. The correlation between BMI and estrogen concentrations in the frontal lobe suggests the importance of non-brain sources of estrogens.

## Introduction

**Figure 1:**
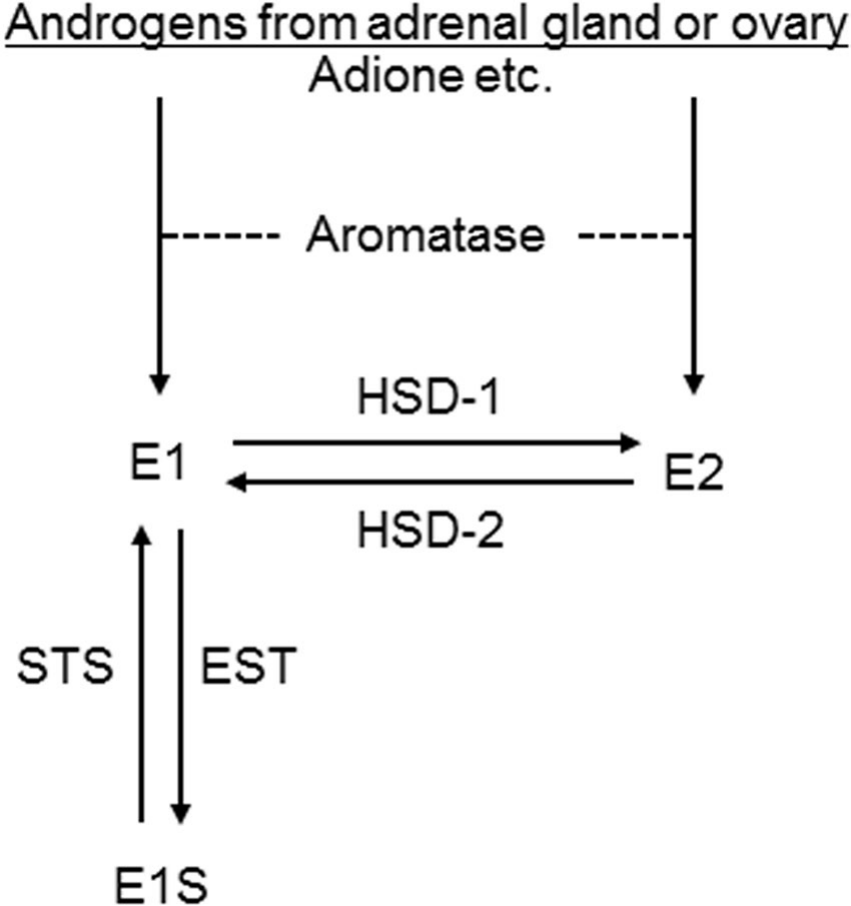
Estrogen-metabolizing enzymes. Aromatase, STS, and HSD-1 act in a direction producing more active estrogens, whereas EST and HSD-2 act in the opposite direction. Adione, androstenedione; E1, estrone; E1S, estrone sulfate; E2, estradiol; STS, steroid sulfatase; EST, estrone sulfotransferase; HSD-1, 17β-hydroxysteroid dehydrogenase type 1; HSD-2, 17β-hydroxysteroid dehydrogenase type 2.

Estrogens play a physiologically important role in various organs, and deficiency/excess of estrogens has been reported to cause various diseases. In the nervous system, estrogens have been reported to maintain homeostasis by protecting neurons against damage, organizing sex-specific neuronal networks, or regulating bioenergetic systems^1–3^. Further, estrogens have been suggested to inhibit amyloid β (Aβ) deposition by favorably regulating β-secretase (BACE1) or neprilysin, which generate and degrade Aβ, respectively^4–7^. Epidemiologically, the risk of Alzheimer’s disease (AD) is higher in women than men, and its incidence in women increases after menopause^8^. Levels of estradiol (E2), a representative and the most potent estrogen, in postmenopausal women reportedly decrease to levels lower than those in men^9^. In some studies at the end of the 1990s, the risk of AD was reported to significantly decrease in women treated with estrogen replacement therapy^10^. These observations suggest a protective effect of estrogens against AD; however, in Women’s Health Initiative (WHI) hormone therapy trials, where the effect of external female hormone use was tested against a placebo, the risk of AD was reported to increase in women treated with hormone (estrogen-progesterone combined) replacement therapy, raising controversies in this study area^11^. Generally, in postmenopausal women, where estrogens from the ovary markedly decrease, peripheral estrogen synthesis via estrogen-metabolizing enzymes (EMEs) is important as the source of estrogens^12^. EMEs include aromatase, steroid sulfatase (STS), estrogen sulfotransferase (EST), and some types of 17β-hydroxysteroid dehydrogenase (HSD). Aromatase converts circulating androgens from the adrenal gland or ovary into estrogens. STS hydrolyzes biologically inactive estrogen sulfates to produce active estrogens. HSD type 1 (HSD-1) catalyzes the 17β-reduction of a biologically weak estrogen, estrone (E1), converting into E2. EST and HSD type 2 (HSD-2) play opposite roles to STS and HSD-1, respectively (Fig. 1)^12^. Examining EMEs as well as estrogen levels in the tissue of interest is important for studying the relationship between estrogens and diseases in postmenopausal women^12^. Further, examination of the expression of estrogen receptors (ERs) in the target tissues is necessary in this study area, because estrogens cannot exert their function in the absence of ERs even if estrogen concentrations are high enough^8, 12^. Although many studies have examined the association between estrogens and AD, few studies have systematically examined estrogen-related factors (e.g. estrogen concentrations, EMEs expression, or ERs expression) in the brain tissues of AD patients. We address this in the present study using the frontal lobe tissues (cortex and white matter, separately) from AD patients and controls. In AD, Aβ deposition or neurofibrillary tangles are typically found in the hippocampus or cerebral cortex, and these are the most frequently studied regions. The frontal lobe is the most suitable for the study of AD using frozen materials for the following reasons: the frontal cortex is frequently affected in AD; the volume of the frontal lobe is large, and separate sample collection from cortex and white matter is straightforward compared with the hippocampus or other cerebral lobes. The white matter, mainly composed of neuronal axons and glias, has been less frequently studied than the cortex in AD; however, we also focused on this, because glias play important roles in maintaining homeostasis of the brain, and increasing evidence has shown their importance in the etiology of AD^13, 14^. Concentrations of E1, E2, and androstenedione (Adione), a representative androgen, were examined in the frontal lobe (cortex and white matter, separately) from AD patients and controls, using liquid chromatography tandem mass spectrometry (LC-MS/MS), the most accurate method for measuring sex steroid concentrations. In addition, expressions of EMEs and two types of ERs, ER-α and ER-β (wild type, ER-β1), were examined by quantitative real-time PCR and immunohistochemistry, respectively. Further, the associations between body mass index (BMI) and steroid concentrations in the frontal lobe were examined, because aromatase is abundantly contained in the fatty tissue, and BMI is reportedly important as a determinant of serum estrogen levels in postmenopausal women^15^.

## Results

### Sex steroids concentrations in the frontal lobe and their association with BMI

**Figure 2:**
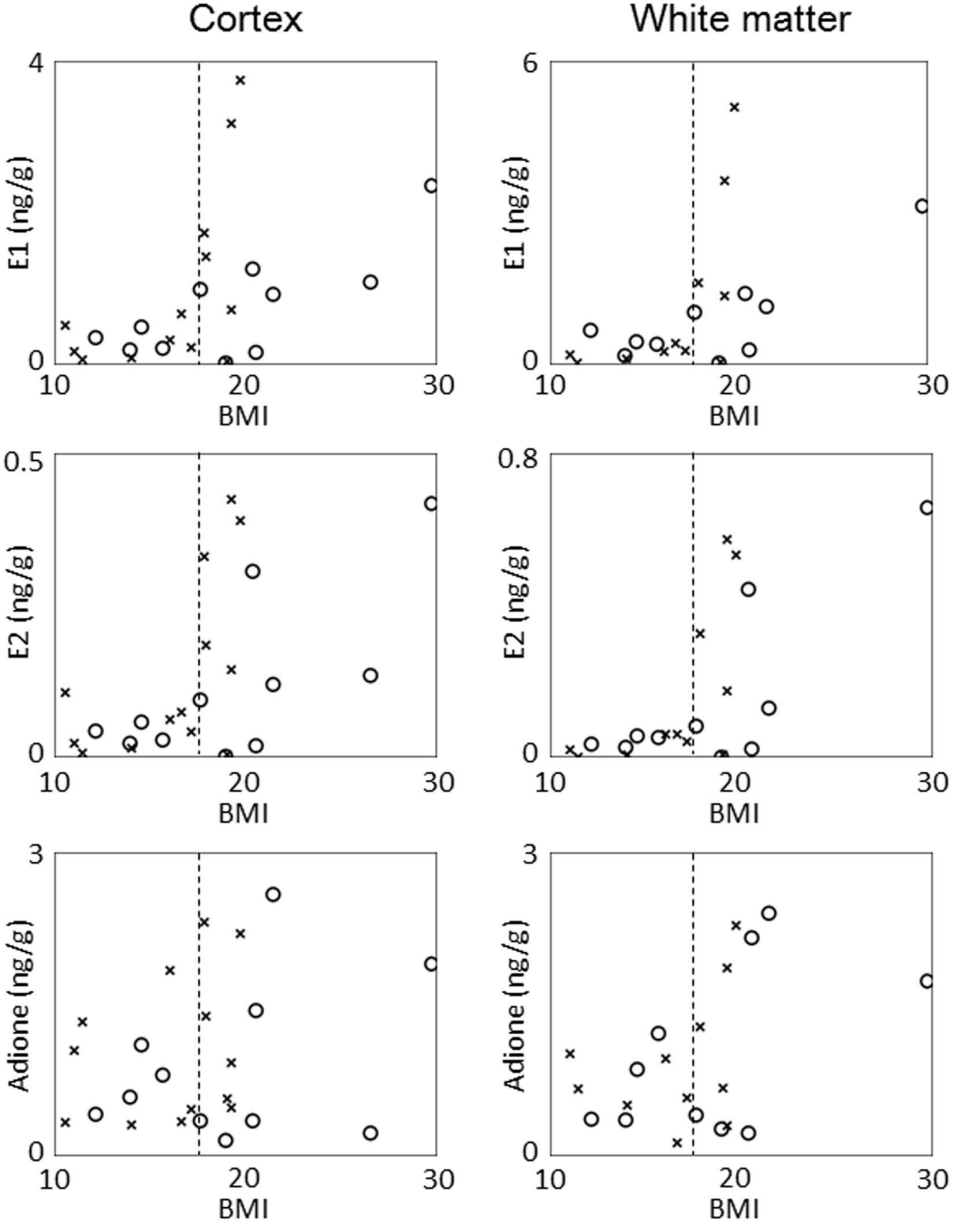
Association between body mass index (BMI) and concentrations of estrone (E1, upper), estradiol (E2, middle), and androstenedione (Adione, lower) in the cortex (left) and white matter (right) of the frontal lobe. ○ and × represent controls and AD patients, respectively. Dotted line, BMI = 17.5.

There was no significant difference in each sex steroid concentration between AD and control groups both in the cortex and white matter (Table 1). BMI in the AD group was lower than that in the control group although statistical significance was not achieved (16.1 vs. 19.3, *P* = 0.0752). The association between each sex steroid concentration and BMI is shown in Fig. 2. Concentrations of E1 and E2 correlated positively with BMI in both the cortex and white matter, with particularly high correlation coefficient in controls (Table 2). The correlation coefficient was lower in the AD group than in controls, with the slope among AD patients being larger for BMI ≥ 17.5 than for BMI < 17.5. Concentrations of E1 and E2 were significantly lower in subjects with BMI < 17.5 than in those with BMI ≥ 17.5 both in the cortex and white matter (Fig. 2 and Table 3). Adione concentration did not present such a clear association with BMI (Fig. 2, Table 2 and Table 3).

**Table 1:** Comparison of concentrations of sex steroid hormones in the cortex and white matter of the frontal lobe between Alzheimer’s disease and control groups.

		AD	Control	*P*-value
Cortex	E1 (ng/g)	0.993 ± 0.281	0.733 ± 0.306	0.5378
	E2 (ng/g)	0.139 ± 0.039	0.112 ± 0.043	0.6574
	Adione (ng/g)	1.039 ± 0.208	0.899 ± 0.226	0.6527
White matter	E1 (ng/g)	1.188 ± 0.415	0.874 ± 0.435	0.6076
	E2 (ng/g)	0.164 ± 0.066	0.150 ± 0.069	0.8852
	Adione (ng/g)	0.931 ± 0.225	0.998 ± 0.236	0.8391

AD, Alzheimer’s disease; E1, estrone; E2, estradiol; Adione, androstenedione.

**Table 2:** Correlation coefficient (r) between body mass index and concentrations of sex steroid hormones in the cortex and white matter of the frontal lobe among Alzheimer’s disease and control groups.

		AD		Control	
		r	*P*-value	r	*P*-value
Cortex	E1	0.565	0.0443	0.764	0.0062*
	E2	0.563	0.0450	0.714	0.0137*
	Adione	0.232	0.4457	0.357	0.2807
White matter	E1	0.623	0.0230	0.815	0.0023*
	E2	0.644	0.0176	0.808	0.0026*
	Adione	0.344	0.2493	0.566	0.0697

^*^Significant; *P* < 0.05/3 (Bonferroni adjustment).

AD, Alzheimer’s disease; E1, estrone; E2, estradiol; Adione, androstenedione.

**Table 3:** Comparison of concentrations of sex steroid hormones in the cortex and white matter of the frontal lobe between subjects with body mass index < 17.5 and ≥ 17.5.

		BMI < 17.5	BMI ≥ 17.5	*P*-value
Cortex	E1 (ng/g)	0.302 ± 0.260	1.358 ± 0.239	0.0068*
	E2 (ng/g)	0.043 ± 0.036	0.198 ± 0.033	0.0043*
	Adione (ng/g)	0.775 ± 0.220	1.143 ± 0.202	0.2305
White matter	E1 (ng/g)	0.301 ± 0.371	1.708 ± 0.354	0.0129*
	E2 (ng/g)	0.036 ± 0.057	0.268 ± 0.054	0.0083*
	Adione (ng/g)	0.664 ± 0.216	1.235 ± 0.206	0.0707

^*^Significant; *P* < 0.05/3 (Bonferroni adjustment).

BMI, body mass index; E1, estrone; E2, estradiol; Adione, androstenedione.

### Expression of EMEs in the frontal lobe

Transcript levels of aromatase, STS, EST, and HSD-1 did not significantly differ between AD and control groups both in either the cortex or white matter. HSD-2 mRNA was undetectable in most samples (Table 4). There was no significant association between each EME mRNA level and any sex steroid concentration. There was no significant association between BMI and each EME mRNA level, either (data not shown).

**Table 4:** Comparison of mRNA level for each estrogen metabolizing enzyme in the cortex and white matter of the frontal lobe between Alzheimer’s disease and control groups.

		AD	Control	*P*-value
Cortex	Arom	2164 ± 1040	729 ± 1083	0.3491
	STS	504 ± 161	929 ± 167	0.0800
	EST	11.73 ± 4.72	8.52 ± 4.91	0.6417
	HSD-1	10.18 ± 2.39	4.50 ± 2.49	0.1135
	HSD-2	UD	UD	—
White matter	Arom	823 ± 394	1477 ± 410	0.2626
	STS	346 ± 306	1272 ± 318	0.0472
	EST	5.65 ± 3.92	6.20 ± 4.08	0.9229
	HSD-1	12.98 ± 6.88	17.73 ± 7.16	0.6363
	HSD-2	UD	UD	—

AD, Alzheimer’s disease; Arom, aromatase; STS, steroid sulfatase; EST, estrone sulfotransferase; HSD-1, 17β-hydroxysteroid dehydrogenase type 1; HSD-2, 17β-hydroxysteroid dehydrogenase type 2; UD, undetected.

### Expression of ERs in the frontal lobe

**Figure 3:**
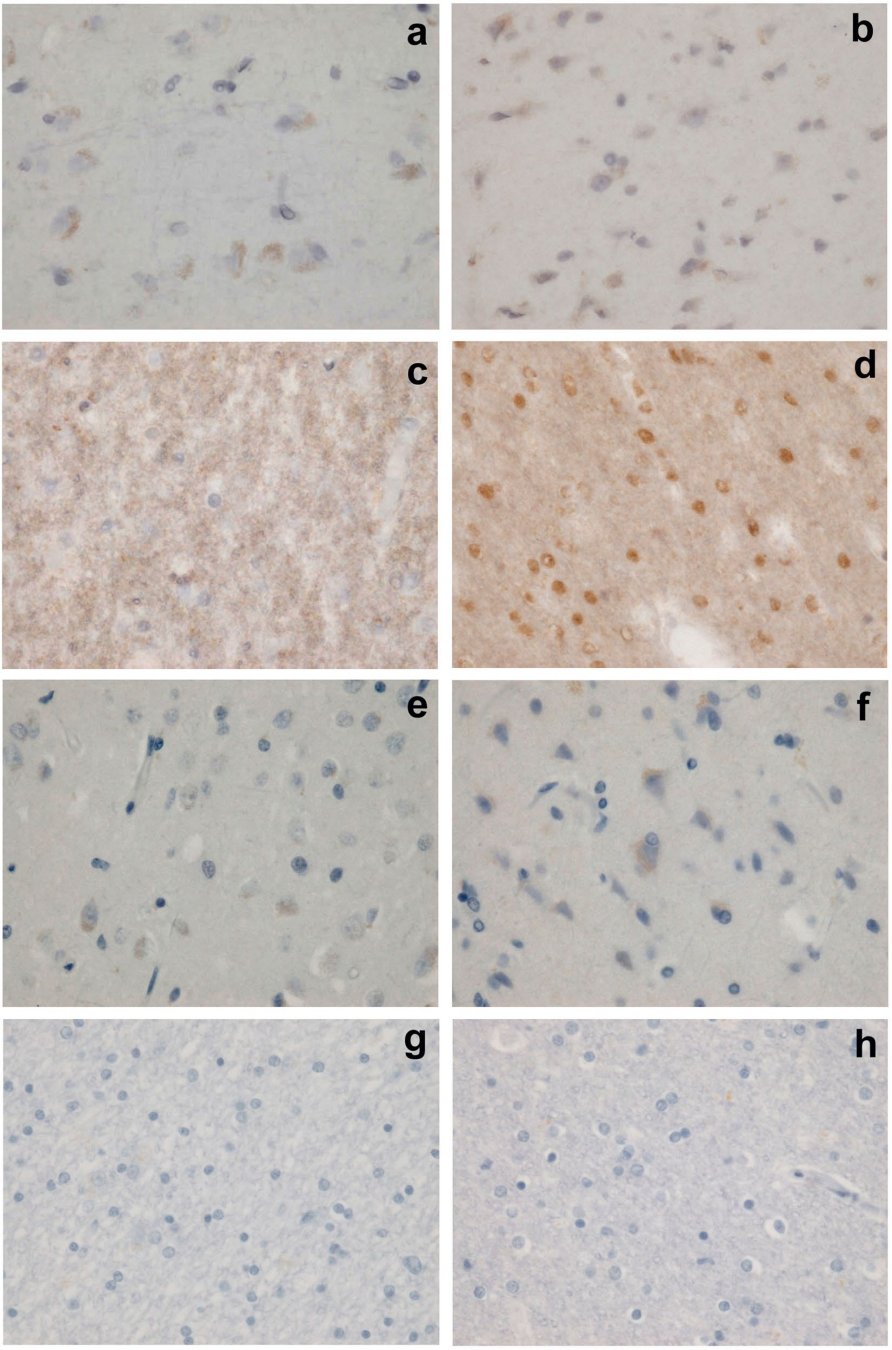
Immunohistochemistry for ER-β1 (**a–d**) and ER-α (**e–h**) in the frontal lobe; cortex from an AD patient (**a,e**) and a control (**b,f**); white matter from an AD patient (**c,g**) and a control (**d,h**).

The Allred score^16–18^ was adopted for assessing expression of ERs, because it is an objective and easy method to semiquantatively estimate ERs in a large area of target tissue. In the white matter, glial nuclear ER-β1 staining was clearly observed for most of the controls, but this was not true for AD patients (Fig. 3c,d). The Allred score for ER-β1 in the white matter was significantly higher in controls than in AD (6.5 ± 0.3 and 3.2 ± 0.3, respectively. *P* < 0.0001). As for ER-α, nuclear staining was not seen in the white matter irrespective of the antibodies used (Fig. 3g,h). In the cortex, the nuclear staining was insufficient to be evaluated irrespective of the ER type or disease status (Fig. 3a,b,e,f).

## Discussion

Estrogen-related factors were systematically examined in the frontal lobe of AD and control groups. There was no significant difference in any of the sex steroid concentrations or expression levels of EMEs between the two groups in either the cortex or white matter, whereas nuclear ER-β1 expression was significantly lower in the white matter from the AD group than in that from controls.

Few studies have systematically examined estrogen-related factors in human brain tissue. Yue *et al*. reported a lower E2 concentration as well as lower aromatase mRNA level in the frontal cortex of AD patients compared with controls. Together with experimental evidence from animal models, they concluded that decrease of aromatase and consequent E2 reduction causes AD^19^. These observations by Yue *et al*. were not reproduced in the present study, although the reason is not known.

The cortex is a representative region affected in AD; however, in the present study, the only factor that was clearly different between the AD and control groups was the decrease of glial nuclear ER-β1 expression (considered to be estrogen-bound ER-β1, as described below) in the white matter from AD patients. White matter is an environment for neurons to interact with glias via neuronal axons. Glias are considered important regulators of neuronal survival/death, because they play important roles in trophic support or maintaining metabolic and ionic homeostasis in neurons, or maintaining synaptic transmission, etc. Recently, Ma *et al*. reported *in vitro* and *in vivo* observations suggesting that estrogens play a neuroprotective role against brain ischemia-reperfusion injury by activating glial cells via ER-β^20^. The participation of glias in the etiology of AD has also attracted attention recently^13, 14^. Furthermore, the importance of ER-β has been suggested in the prevention of Aβ accumulation and plaque formation, and the selective activation of ER-β has been suggested as a promising treatment for AD^7, 8, 21^. Taking our findings together with these reports, we assume that estrogen-bound ER-β in glias in the white matter exerts a neuroprotective action against AD development through glial-axonal interaction. Nuclear ER-β loss in glias, suggesting neuroprotective dysfunction, may lead to the neuronal loss observed in the AD cortex. The possibility that ER-β loss in glias is the result, not the cause, of AD cannot be ruled out at present. *In vitro* and *in vivo* studies taking not only neuronal cells but also glial cells into account are needed to clarify the importance of glial ER-β loss in the etiology of AD. Although there have been limited number of studies immunohistochemically examining ER (ER-α and/or ER-β) expression in the brain from AD patients, no consensus has been obtained thus far whether ERs decrease or increase in AD^22–27^. This is at least partly because of the diversity of the study settings. The brain is composed of many parts and various cell types having different functions. It is no wonder that the results are different among studies according to the parts or cell types examined. Results may also differ according to which of the intracellular structures are examined. Cytoplasmic staining has been estimated in most studies: however, we did not evaluate cytoplasm, because it was difficult to appropriately and differentially evaluate cytoplasm for the variety of cell types present in the frontal lobe. Although the importance of ERs in cytoplasm or cell membrane has recently been recognized, ERs are primarily nuclear receptors and nuclear staining is the first in line to be estimated^8, 16–18^. Like other nuclear receptors, ER localizes to the nucleus on binding with estrogens; therefore, nuclear ER expression suggests that the ER is estrogen-bound and activated/functional. A group from the Netherlands examined nuclear ER expression in AD patients’ brains^22, 26, 27^. Increased nuclear ER-α and ER-β expression was reported in the neurons of the nucleus basalis of Meynert in AD patients^26^. Higher ER-α nuclear expression in AD patients than in controls was reported in the infundibular nucleus of the hypothalamus^22^. To our knowledge, the present study is the first to have focused on nuclear ER expression in the white matter of the frontal lobe, and to have shown a decrease in ER-β expression in AD patients. Differences in immunohistochemical methods may also cause different results. We used a method established in clinical practice/study for breast cancer using robust antibodies^16–18^; however, most studies in the nervous system used other antibodies. In the present study, two kinds of antibodies were tried for ER-α, but neither yielded nuclear staining.

One of the most novel and important findings in the present study is that estrogen concentrations exhibited strongly positive correlation with BMI but not with aromatase mRNA level in the frontal tissue from controls. This suggests that non-brain sources are important as determinants of estrogen concentrations in the frontal lobe. BMI is known to positively correlate with serum estrogen levels in postmenopausal women, because aromatase is abundantly contained in the adipose tissue^15^. Estrogens and androgens have a steroidal structure and are highly lipophilic. Serum steroids may easily transfer and accumulate in the brain which is abundant in lipid, resulting in a close correlation between serum and frontal steroids concentrations, finally leading to a correlation between BMI and frontal estrogen concentrations. Interestingly, the correlation coefficient between BMI and frontal estrogen concentrations observed in the control group (0.714–0.815, Table 2) is higher than that reported for serum estrogens (0.52–0.60)^15^, suggesting a major effect of BMI on frontal estrogens. The lower correlation coefficient in AD than in the control group may reflect a rapid BMI decrease in AD patients, which has been reported in many studies^28–30^. The causal relationship between BMI and AD is not fully understood, but the following hypotheses have been suggested: eating disorders in AD (or pre-AD) patients may cause BMI decrease, or decreased BMI resulting in estrogen reduction may cause AD. In the present study, BMI 17.5 seems to be somewhat of a threshold for maintaining estrogen concentrations in the frontal lobe. This association between AD and BMI decrease may lead to a vicious cycle for the maintenance of brain homeostasis mediated by estrogens.

The concentrations of E1 and Adione in the frontal lobe observed in the present study were several times higher, while that for E2 was several score times higher, than those in the postmenopausal serum reported in other studies^31^. The higher concentration of E1 and Adione in the frontal tissue than the serum can be mostly ascribed to their lipophilia, but this is unlikely for E2′s remarkably higher concentration. In the present analyses of EMEs, HSD-2 was undetectable in most cases, while HSD-1 was detectable for almost all cases. The relative dominance of HSD-1 over HSD-2 would accelerate the conversion of E1 into E2, leading to marked condensation of E2 in the frontal tissue (Fig. 1)^12^. Such a high level of estrogens concentrations in the frontal lobe, together with distinct nuclear ER-β1 expression in glias among controls, may add further evidence for the physiologically important role of the estrogen signaling system in the brain. A compensatory mechanism, or lack thereof, to impairment of the estrogen signaling system may determine the risk of AD^3^.

In conclusion, in a systematic analysis examining estrogen-related factors for the cortex and white matter of the frontal lobe from AD and control groups, the only remarkable difference between the two groups was a marked decrease of glial nuclear ER-β1 expression in the white matter from the AD group, suggesting the necessity of studying the effect of estrogens on glias as well as neurons in the etiology of AD. Frontal estrogen concentrations were closely correlated with BMI, suggesting that extremely low BMI is not favorable for maintaining estrogen concentrations in the brain.

## Materials and Methods

### Patients

Brain tissues (convex of the second frontal gyrus) were obtained from 13 pathologically confirmed Alzheimer’s disease patients and 12 age adjusted controls. All of them were Japanese women autopsied at the Tokyo Metropolitan Geriatric Hospital between 1999 and 2006. The study protocol was approved by the ethics committee of Tokyo Metropolitan Geriatric Hospital and Fujita Health University. All methods were performed in accordance with the relevant guidelines and regulations. Written informed consent, including consent to use in medical studies, was obtained from the bereaved family of each patient prior to autopsy.

### Quantitative Analysis by LC-MS/MS

Concentrations of E1, E2 and Adione were measured using LC-MS/MS at ASKA Pharma Medical Co., Ltd (Kawasaki, Japan). Briefly, weighed brain tissues (40–200 mg) were homogenized, and each internal standard (100 pg of each internal standard, E1-^13^C_4_, E2-^13^C_4_, and Adione-d7) and ethanol were added, and then shaken at 50 °C for 2 hrs. The obtained extracts were applied to a Bond C18 cartridge column (Varian, Harbor City, CA), and the steroid fraction was eluted with 80% acetonitrile. The steroids fraction was loaded onto a mixed-mode cartridge (Oasis MAX, Waters, Milford, MA) to separate the neutral and phenol fractions containing androgens and estrogens, respectively. Estrogens were measured by LC-MS/MS, an API-5000 triple stage quadrupole mass spectrometer (Appied Biosystems, Foster City, CA) connected to an LC-20AD pump and SI HTC autosampler (Shimadzu, Kyoto, Japan), and electrospray ionization ion source devices, using the column, a Xterra MS C_18_ (2.1 mm × 100 mm I.D.3.5 μm; Waters) at 40 °C. Adione was measured by an LC-MS/MS instrument, API-4000 (Appied Biosystems) equipped with an ESI ion source and an Agilent 1100 HPLC system (Agilent Technologies, Santa Clara, CA) with an HTC PAL auto-sampler (CTC Analytical, Zwingen, Switzerland), using the column, Cadenza CD-C_18_ (150 mm × 3 mm I.D., 3 μm; Imtakt, Kyoto, Japan) at 40 °C.

### Determination of EMEs mRNA level

Frozen brain tissues (10–50 mg) were smashed to fine powders by Micro Smash MS-100R (TOMY SEIKO Co., Tokyo, Japan) with stainless beads, SUB-55 and zirconia beads, ZB-10 (TOMY SEIKO Co.) at 4 °C and extracted with 1 ml of TRIzol Reagent (Invitrogen, Carlsbad, CA) to prepare total RNA according to manufacturers’ instructions. The cDNA was synthesized from the total RNA by SuperScript II reverse transcriptase (Invitrogen) using random hexamers (Applied Biosystems). The resulting cDNA was subjected to quantitative PCR reactions using TaqMan probes (Applied Biosystems). Transcript levels of EMEs were determined using the following TaqMan Gene Expression Assays (TaqMan probes): Hs00903413_m1 (aromatase), Hs00165853_m1 (STS), Hs00193690 (EST), Hs00166219_m1 (HSD-1), Hs00157993_m1 (HSD-2), and Hs99999903_m1 (β-actin). The fluorescence generated by cleavage of the TaqMan probes during PCR was analyzed by a 7300 Real Time PCR system (Applied Biosystems). Briefly, a total of 20 μl reaction mixture consisting of 10 μl TaqMan Gene Expression Master Mix (Applied Biosystems), 1 μl Primer/TaqMan probe, and the cDNA was amplified by PCR (40 cycles of 95 °C for 10 sec and 60 °C for 30 sec). The level of each EME transcript was calculated from the respective PCR cycle threshold (Ct) value by ΔΔCt method.

### Immunohistochemical examination of ERs status

Immunostaining was performed for sections of formalin-fixed and paraffin-embedded tissue according to the methods used for breast cancer samples as described elsewhere^18^. ER-α and ER-β1 were detected by anti-ER-α mouse monoclonal antibodies (clone 1D5; Dako, Glostrup, Denmark and clone 6F11; Novocastra, Newcastle upon Tyne, England) and anti-ER-β1 mouse monoclonal antibody (clone PPG5/10; Dako) specific for ER-β1, respectively^16–18^. As there is no standard method for assessing ER expression in the nervous system, the Allred score routinely used in clinical practice for breast cancer was adopted for evaluation. Briefly, nuclear immunoreactivity for ER-α and ER-β1 is estimated independently by summing the percentage score, PS, and intensity score, IS, of positively stained cells (PS: 0%, 0; <1%, 1; <10%, 2; <33%, 3; <67%, 4; ≥67%, 5. IS: weak, 1; medium, 2; strong, 3)^17^.

### Statistical analysis

The t-test was used to compare concentrations of each steroid hormone, BMI, mRNA level of each EME, or Allred score for each ER, between AD and control groups. In all instances, the statistical software JMP 12.0.1 (SAS Institute, Cary, NC) was used. *P* < 0.05, when necessary dividing by the number of factors examined (Bonferroni adjustment), was considered significant.
